# COULD CHASING GRANDKIDS KEEP US YOUNG? THE ASSOCIATION BETWEEN CUSTODIAL GRANDPARENT STATUS AND HEALTH

**DOI:** 10.1093/geroni/igz038.2477

**Published:** 2019-11-08

**Authors:** Danielle K Nadorff, Emily A Williamson, Ian T McKay

**Affiliations:** 1 Mississippi State University, Starkville, Mississippi, United States; 2 Mississippi State University, Mississippi State, Mississippi, United States

## Abstract

There are approximately 7.2 million grandparents living with their grandchildren in the United States. Of these, roughly 2.5 million are skipped-generation households in which grandparents are solely responsible for meeting the needs of their grandchildren (U.S. Census, 2017). Previous research has established that custodial grandparents suffer from added strain and burden compared to their peers, which negatively impacts their health (Lo, M., Liu, Y., 2009). A decline in functional ability has a negative impact on not only the lives of these older adult grandparents but also their family members who are dependent upon them for care. The current study examines adults aged 65 and older using data from the American Community Survey 2016 to assess the extent to which raising one’s grandchildren is associated with five areas commonly subject to decline in older adulthood: cognitive performance, self-care ability, ambulatory difficulty, hearing, and vision abilities. Hierarchical binary logistic regression analyses found that after controlling for the effects of age, sex, race, and income-to-poverty ratio, custodial grandparents (those who reported having primary responsibility for their grandchildren in their own home with no parents present) were significantly less likely than their peers to report experiencing any of these five disabilities. Details of each model and clinical implications will be discussed.

